# Evaluation of the Expression and Function of the *TRE2-like* and *TRE2* Genes in Ecdysis of *Harmonia axyridis*

**DOI:** 10.3389/fphys.2019.01371

**Published:** 2019-11-01

**Authors:** Yan Li, Xu Chen, Sha-Sha Wang, Bi-Ying Pan, Shi-Gui Wang, Su Wang, Bin Tang

**Affiliations:** ^1^College of Life and Environmental Sciences, Hangzhou Normal University, Hangzhou, China; ^2^Institute of Plant and Environment Protection, Beijing Academy of Agriculture and Forestry Sciences, Beijing, China

**Keywords:** *Harmonia axyridis*, physiological activities, RNA interference, *TRE2-like*, *TRE2*

## Abstract

*Harmonia axyridis* is an important predatory insect and widely used in biological control of agricultural and forestry pests. Trehalose is directly involved in the energy storage of the *H. axyridis* and in the oxidative function of various physiological activities thereby providing an energy source for its growth and development. The aim of this study was to explore the potential function of membrane-bound-like trehalase (*TRE2-like*) and membrane-bound trehalase (*TRE2*) genes in *H. axyridis* by RNAi. In addition, the activity of soluble and membrane-bound trehalase and the expression of genes related to trehalose and glycogen metabolism were determined in the larvae injected with ds*TRE2-like* or ds*TRE2*. The results showed that wing abnormality and mortality appeared in adults, as well as the activity of soluble trehalase and glycogen contents increased when interfering with *TRE2-like* gene. However, the activity of membrane-bound trehalase, trehalose and glucose contents in the larvae decreased. The expression of glycogen synthase (*GS*) and glycogen phosphorylase (*GP*) genes were decreased after RNAi in the ecdysis stage. The expression of chitin synthase gene A (*CHSA*), chitin synthase gene B (*CHSB*), and trehalose-6-phosphate synthase genes (*TPS*) were decreased significantly after RNAi, especially in the ecdysis stage. These results indicated that RNA interference is capable of knocking down gene expression of *TRE2-like* and *TRE2*, thereby disrupting trehalose metabolism which affects the chitin synthesis pathway in turn and also leads to developmental defects, such as wing deformities. This study could provide some theoretical guidance for the function of *TRE2* gene in other insects.

## Introduction

*Harmonia axyridis* (Pallas) (Coleoptera: Coccinellidae) is an important natural enemy of aphids and other pests (Nakayama et al., 2010; Wu et al., 2016; Adachi-Hagimori et al., 2019). The typical breeding strategy of *H. axyridis* involves multiple mating and thus increasing its population (Awad et al., 2017; Du et al., 2019). When faced with various natural threats, *H. axyridis* adapts with different strategies, such as gathering in winter to protect against cold (Durieux et al., 2015) and releasing harmful exudates to expel natural enemies (Schmidtberg et al., 2019). As an immediate source of energy, trehalose plays a vital role in the energy storage of the *H. axyridis*, in the oxidative function of various physiological activities, and in the growth and development (Ge et al., 2011; Xie et al., 2013; Kojić et al., 2018; Pathak et al., 2018; Zhao et al., 2018). Trehalose is called the “blood sugar of insects” (Yu et al., 2008; Wu et al., 2019), and it is a non-reducing disaccharide composed of glucose units linked by two glycosides and is found in bacteria, yeasts, fungi, plants, insects, and other invertebrates (Elbein et al., 2003; Frison et al., 2007; Tang et al., 2008). Trehalose is widely distributed in different tissues and expressed in various development stages of insects, and mainly synthesized in the fat body of insects and released into the hemolymph rapidly (Thompson, 2003).

The decomposition of trehalose is catalyzed by a specific α-glucosidase-trehalase (TRE) (Tang et al., 2018). Trehalase hydrolyzes the α-1, 1-glycosidic linkage of trehalose to release two glucose molecules (Shukla et al., 2015; Łopieńska-Biernat et al., 2019). Trehalase was discovered in *Aspergillus niger* in 1893 and was classified into two types, namely, soluble trehalase (TRE1) and membrane-bound trehalase (TRE2) (Forcella et al., 2010; Ma et al., 2015). It has been identified in several insect species, such as *Apis mellifera* (Lee et al., 2007), *Aphis glycines* (Bansal et al., 2013), *Spodoptera exigua* (Tang et al., 2008; Zou et al., 2013), *Tribolium castaneum* (Parkinson et al., 2003; Tang et al., 2016), *Nilaparvata lugens* (Zhao et al., 2016), and *Chironomus ramosus* (Shukla et al., 2018). The first identified trehalase in insects was soluble trehalase cloned in *Tenebrio molitor* (Takiguchi et al., 1992). In addition, TRE2 was first discovered in silkworm in 2005 (Mitsumasu et al., 2005). Studies have shown that trehalase regulates insect homeostasis and development, and plays an important role in insect ecdysis (Chen et al., 2010; Tan et al., 2014).

TRE2 is an exogenous transmembrane enzyme involved in the hydrolysis of exogenous trehalose to provide energy (de Almeida et al., 2009). Some TRE2 proteins are present in an inactive state, but when activated they can destroy membrane integrity (Wegener et al., 2003). These are mainly present in the mitochondria as well as in the brain, stratum, cuticle, and midgut, with active sites outside the cell (Tang et al., 2008). In insects, TRE2 is involved in several physiological processes such as flight, reproduction, development, and digestion (Wegener et al., 2010). *TRE2* gene has been cloned in a variety of insects, mainly in the fat body, midgut, and Malpighian tube. Gene expression patterns have shown differences among insect species suggesting a potential functional diversification of the trehalase enzymes during their evolution (Nardelli et al., 2019). There might be only two kinds of trehalases in insects, however, a membrane-bound-like trehalase (TRE2-like) that is a class of membrane-free proteins with high homology to membrane-bound proteins has been reported in *Locusta migratoria manilensis* (Liu et al., 2016). *TRE2-like* is likely to be an intermediate type of soluble trehalase, which is similar to the *TRE2*, with dominant and negative forms reported by Wegener et al. (2003), but *TRE2-like* functional role is still unclear. Trehalose is catabolized by trehalase, and it regulates energy metabolism and glucose production, which is a concern to be addressed in insect physiology (Zhang et al., 2019b). Studies on the function of *TRE1* gene homologs are common, but studies on the specific functions of homologous genes of *TRE2* are rare. Moreover, the action mechanisms of *TRE2-like* and *TRE2* genes in insects remain unclear and little is known about their structure, tissue distribution, and expression patterns. Therefore, it is necessary to further understand and explore their functions.

As *TRE* genes are known to be vital to insect growth and development (Zhang et al., 2019a), the present study investigated the functions of *TRE2-like* and *TRE2* genes in *H. axyridis* using RNAi via the injection of double-stranded RNA (dsRNA) corresponding to conserved *TRE2-like* and *TRE2* genes structure. In this study, we aimed to synthesize dsRNA fragments from the conserved domains of *TRE2-like* and *TRE2* genes from the *TRE* sequence of *H. axyridis*, analyzed the expression and function of *TRE2-like*, and *TRE2* in the third and fourth instar larvae of *H. axyridis*. The results will elucidate the trend in gene expression, explain the metabolic mechanism, and provide a theoretical basis for exploring the function of *TRE2-like* and *TRE2* genes.

## Materials and Methods

### Insects

*Harmonia axyridis* individuals were raised in the Key Laboratory of Animal Adaptation and Evolution of Hangzhou Normal University. They were maintained in an artificial climate chamber at 25 ± 1°C, 70 ± 5% relative humidity, and 14:10 h photoperiod. *H. axyridis* individuals were fed *Aphis medicaginis* (Homoptera, Aphididae) at a fixed time every day. The third and fourth instar larvae of *H. axyridis* were collected for microinjection; they were analyzed 48 h after injection and during ecdysis. About one hundred individuals were injected per treatment. Then, 3 to 5 individuals were selected from each injection group for Total RNA isolating which were repeated three times.

### Total RNA Isolation, dsRNA Synthesis, and Treatments

TRIzol (Invitrogen, Carlsbad, CA, United States) was used to extract total RNA from *H. axyridis*. The total RNA integrity was determined by 1% agarose gel electrophoresis. The concentration and purity of the total RNA were determined using the NanoDrop 2000 spectrophotometer (Thermo Fisher Scientific, Waltham, MA, United States). The cDNA template was synthesized according to the instruction manual of the PrimeScript^®^ RT Reagent Kit (NARISHIGE, JAPAN) and gDNA Eraser Reverse Transcription Kit; the reverse transcription product was stored at −20°C. According to the *TRE2-like* (KX349224.1, 2,133 bp, Supplementary Image S1) and *TRE2* (KX349225.1, 2,374 bp, Supplementary Image S2) genes coding regions of *H. axyridis*, specific primers with the T7 promoter sequence in the 5′ end were designed. Based on the cDNA of *H. axyridis* (Table 1), fragments of the *TRE2-like* and *TRE2* genes were amplified by real-time polymerase chain reaction (RT-PCR). The PCR conditions were as follows: 40 cycles at 95°C for 30 s, 58°C for 30 s, and 72°C for 45 s, and finally extended at 72°C for 10 min. The purified *TRE* amplicon synthesized *in vitro* was used to synthesize dsRNA using the T7 RiboMAX Express RNAi System (Promega Corporation, Madison, WI, United States) (Zhao et al., 2016). The samples were DNase and RNase free. The integrity of the dsRNA was detected by 1.5% agarose gel electrophoresis, and the concentration was determined using a micro spectrophotometer (Narishige, Japan). Moreover, the dsRNA of high quality was stored at −20°C. ds*TRE2-like*, ds*TRE2*, a mixture of ds*TRE2-like* and ds*TRE2*, and the control ds*GFP* were injected into the abdomen of the third and fourth instar of *H. axyridis* using the IM-31 microinjector (Narishige, Japan), and the injection amount of dsRNA was approximately 300 ng per individual. The larvae were sampled at 48 h after injection or during ecdysis.

**TABLE 1:** Primers used for double stranded RNA synthesis.

**Primer name**	**Primer sequence**
DS*HaTRE2-like*-F	CAGGTGGGAGATTCAGG
DS*HaTRE2-lik*e-R	TCAATGTAGGAGGCTGTG
DS*HaTRE2*-F	CCCAAGGACTGGATAAG
DS*HaTRE2*-R	CAATAAAGGTGGTTGAGAA
DS*HaGFP*-F	CCTGAAGTTCATCTGCACCA
DS*H*a*GFP*-R	ACAAGCAGAAGAACGGCATCA

**T7 sequence: GGATCCTAATACGACTCACTATAGG*.*

### Detection of the Relative Expression Level of Key Genes Involved in Carbohydrate Metabolism by qRT-PCR

The total RNA from treated *H. axyridis* larvae was extracted as a template, and specific primers (Table 2) were used for quantitative RT-qPCR. The relative mRNA expression level of *TRE2-like* or *TRE2* was evaluated by qRT-PCR using the SYBR Green master mix (SYBR Green Premix Ex Taq, Takara, Japan) with the Bio-Rad CFX96TM Real-Time PCR Detection System (Bio-Rad Laboratories Inc.). Three biological replicates and three technical replicates were set for each treatment group. The total reaction mixture of volume 20 μL comprised of 10 μL of SYBR Green, 1 μL each of forward and reverse primers (10 μmol/L), 2 μL of cDNA template, and 6 μL of ddH_2_O. The PCR amplification conditions were as follows: pre-denaturation at 95°C for 30 s, denaturation at 95°C for 5 s, and extension at 60°C for 20 s, 40 cycles. The dissolution curve was drawn after each reaction to ensure no non-specific amplification.

**TABLE 2:** Primers used for qRT-PCR.

**Gene**	**Forward (5′–3′)**	**Reverse (5′–3′)**
RT*HaTRE2-like*	TTCCAGGTGGGAGATTCAGG	GGGATCAATGTAGGAGGCTGTG
R*THaTRE2*	CAATCAGGGTGCTGTAATGTCG	CGTAGTTGGCTCATTCGTTTCC
RT*HaTRE1-1*	CTTCGCCAGTCAAACGTCA	CCGTTTGGGACATTCCAGATA
RT*HaTRE1-2*	TGACAACTTCCAACCTGGTAATG	TTCCTTCGAGACATCTGGCTTA
RT*HaTRE1-3*	ACAGTCCCTCAGAATCTATCGTCA	GGAGCCAAGTCTCAAGCTCATC
RT*HaTRE1-4*	TTACTGCCAGTTTGATGACCATT	CATTTCGCTAATCAGAAGACCCT
RT*HaTRE1-5*	TGATGATGAGGTACGACGAGAA	GTAGCAAGGACCTAACAAACTGC
RT*HaTPS*	GACCCTGACGAAGCCATACC	AAAGTTCCATTACACGCACCA
RT*HaGS*	CCCTTAGGATCGGATGTTCTC	CACCAGCCATCTCCCAGTT
RT*HaGP*	GCTGAAGCCCTCTACCAACT	CGCCGTACTCGTATCTTATGC
RT*HaGHSA*	TGCCTCCTACTTCGCCTAC	CTGGGATGGTGAGATTGACA
RT*HaGHSB*	TCCAATGCTCAACACCTACG	CTCTGATGACGCCTTACCAA
Q*Ha-rp*49	GCGATCGCTATGGAAAACTC	TACGATTTTGCATCAACAGT

### Determination of Key Enzyme Activity and Carbohydrate Content During Ecdysis After RNAi in *H. axyridis*

The activities of soluble trehalase, membrane-bound trehalase and the content of sugars were analyzed in *H. axyridis* larvae sampled at 48 h or during ecdysis after injection. Three individuals were selected from each injection group for this part of the experiment. The *TRE* activity assay was performed according to a previously published method with some modifications (Tatun et al., 2008). Briefly, insect samples were ground with phosphate buffered saline (PBS), sonicated, and centrifuged at 4°C for 20 min at 10,000 g. Subsequently, 350 μL of the supernatant was collected and ultracentrifuged at 20,800 g for 60 min at 4°C. The remaining supernatant was used to determine the protein, trehalose, and glycogen contents. The supernatant obtained from ultracentrifugation was used to determine the TRE1 activity. The pellet was resuspended in PBS (pH 7.0) and used to evaluate the TRE2 activity.

The contents of protein and glucose in the supernatant and pellet were determined. Briefly, 75 μL of 40 mM trehalose (Sigma-Aldrich, Saint Louis, MO, United States) and 165 μL of PBS (pH 7.0) were added to the supernatant (60 μL) to determine the TRE activity. The mixture was incubated at 37°C for 60 min and centrifuged at 12,000 g for 10 min at 4°C. The supernatant (50 μL) was used to measure the TRE activity using the glucose (Go) assay kit (Sigma-Aldrich). The protein content was determined using the BCA Protein Assay Kit (Beyotime, China).

The trehalose content was measured using the anthrone method following a previously published method (Leyva et al., 2008). Briefly, 30 μL of 1% sulfuric acid was added to 30 μL of the sample and incubated at 90°C for 10 min and placed in an ice bath for 3 min. After adding 30 μL of 30% KOH, the sample was incubated again at 90°C in a water bath for 10 min and in an ice bath for 3 min. Then, 600 μL of developer (0.02 g fluorenone + 100 mL of 80% H_2_SO_4_) was added, and the sample was placed in a water bath at 90°C for 10 min and cooled in an ice bath. The absorbance of the sample was measured at 630 nm using a microplate reader.

For glycogen content determination, 160 μL of supernatant obtained after centrifugation at 1,000 g was added to 600 μL of anthrone sulfate reagent, and the mixture was incubated at 90°C for 10 min, and then cooled in an ice bath. The absorbance of the sample was measured at 625 nm using a microplate reader. A glucose assay kit (SIGMA) was used to measure the glucose content. Briefly, 150 μL of supernatant obtained after centrifugation at 20,800 g and the suspension of precipitation obtained after centrifugation at 20,800 g were added to an Eppendorf tube, and then 300 μL of glucose analysis reagent was added to the sample, which is the same as the glucose standard sample. After incubation in water bath at 37°C for 30 min, 300 μL of 2 N H_2_SO_4_ was added to stop the reaction, and the absorbance of the samples was measured at 540 nm using a microplate reader.

### Statistical Analyses

In the early stage, we tested the specificity and validity of the primers used in this experiment. The results showed that the dissolution curves were all single peaks, and the peak position was the set annealing temperature, indicating that the specificity of the amplification products was good and the experimental results were effective. In addition, GFP was used as a control for each plate in this experiment, and three biological replicates and three technical replicates were set for each treatment group. The qRT-PCR data were processed using the 2^–ΔΔCT^ method (Livak and Schmittgen, 2001). The mRNA expression level in the dsGFP-injected groups was designated as controls. Statistical analysis was performed with STATISTICA 8.0 and Sigma Plot 10.0, all of the data obtained in this study are presented as means ± standard errors (SEs) of were analyzed using one-way analysis of variance (ANOVA) and Tukey’s test. A *P*-value of 0.05 or 0.01 was considered significant or highly significant, respectively. An asterisk indicates a statistically significant difference in mRNA levels between the dsGFP injection group and each of the dsTRE injection groups measured at the same time (*P* < 0.05, *T-*test), and a double asterisk indicates a highly significant difference (*P* < 0.01,*T-*test).

## Results

### Analyses of Phenotypes, Aberrations and Mortality Rates After *TRE2-like* and *TRE2* Gene RNAi

**FIGURE 1:**
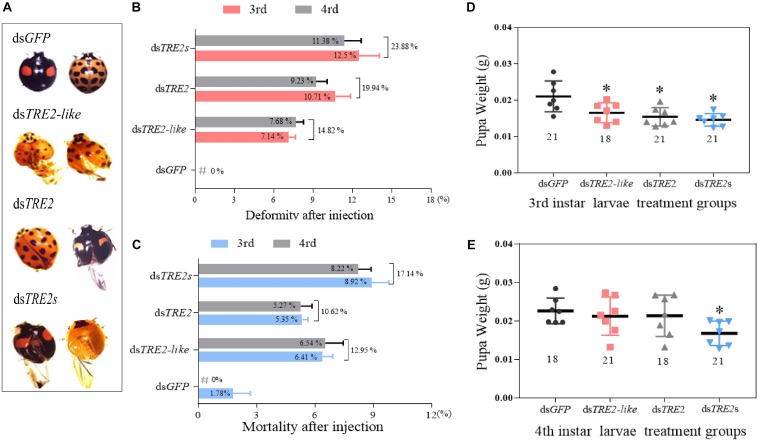
Changes in the wing phenotype, mortality, and body weight after RNAi. **(A)** Larval phenotypes after ds*TRE2-like*, ds*TRE2*, and ds*TRE2*s injection. They were taken under a Leica microscope (16X). **(B)** Phenotypic deformity rates after *TRE2-like*, ds*TRE2*, ds*TRE2*s, and ds*GFP* injection. **(C)** Mortality rates after *TRE2-like*, ds*TRE2*, ds*TRE2*s, and ds*GFP* injection. **(D)** The average pupal weight after the injection of ds*TRE2-like*, ds*TRE2*, and ds*TRE2*s. The third instar larvae were chosen as the targets for dsRNA injection. **(E)** The average pupal weight after the injection of ds*TRE2-like*, ds*TRE2*, and ds*TRE2*s. The third instar larvae were chosen as the targets for dsRNA injection. ^∗^*P* < 0.05 and ^∗∗^*P* < 0.01. Green fluorescence protein (*GFP*) was used as the control (Eighteen or twenty-one individuals were selected from each injection group for phenotypic observation and pupal weight measurement, which was repeated three times).

With the successful silencing of the *TRE2-like*, *TRE2*, and *TRE2s* genes, the adult *H. axyridis* exhibited wing abnormality (Figure 1A). About 14.82% of ds*TRE2-like*, 19.94% of ds*TRE2* and 23.88% of ds*TRE2s* wing deformities were found in the adult *H. axyridis* due to RNAi knockdown (Figure 1B). After *TRE2-like*, *TRE2*, and *TRE2s* were silenced by RNAi, the mortality rate of axyridis larvae was 17.14, 10.62, and 12.95%, respectively (Figure 1C). The aberration and mortality rates of *H. axyridis* injected with control dsRNA against *GFP* were only 0 and 1.78%, respectively (Figures 1B,C). Furthermore, the pupa weights with injection of ds*TRE2-like*, ds*TRE2*, and ds*TRE2s* at the beginning of third instar were significantly decreased (*P* < 0.05) (Figure 1D). After the injection of ds*TRE2-like* and ds*TRE2* to the fourth instar larvae, the pupal weights were decreased, but not significant. The weight loss of pupae after the injection of ds*TRE2s* was significant (*P* < 0.05) (Figure 1E).

### *TRE2-like* and *TRE2* mRNA Expression After RNAi

**FIGURE 2:**
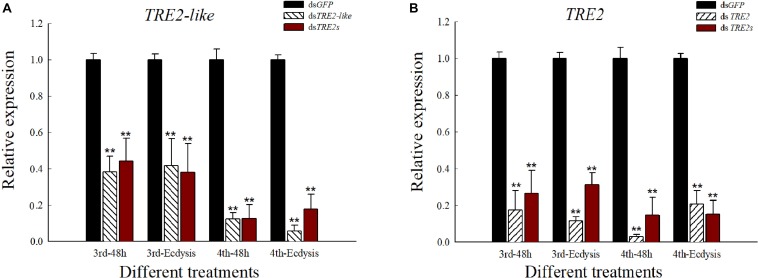
Expression level of *TRE2-like* and *TRE2* in *H. axyridis* after RNAi. **(A)** The expression level of *TRE2-like* gene after the injection of ds*TRE2-like* and ds*TRE2s*. **(B)** The expression level of *TRE2* after the injection of ds*TRE2* and ds*TRE2*s. The third and fourth instar larvae were chosen as the targets for dsRNA injection, ^∗^*P* < 0.05 and ^∗∗^*P* < 0.01.

The mRNA levels of the relevant genes after RNAi was determined by qRT-PCR. All transcripts of the *TRE2-like* and *TRE2* genes were significantly decreased (*P* < 0.01) compared with the ds*GFP* injected treatment after 48 h or ecdysis. The altered trend after the injection of ds*TRE2s* was similar to that of single gene silencing (Figures 2A,B).

### The Activity of Soluble Trehalase and Membrane-Bound Trehalase Changed After *TRE2-like* and *TRE2* Genes RNAi

**FIGURE 3:**
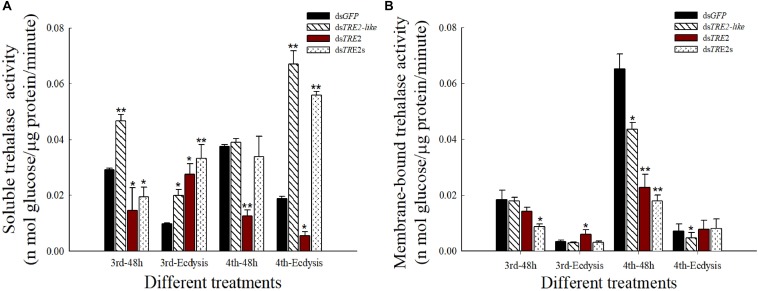
Effects of RNAi on the activity of soluble trehalase and membrane-bound trehalase in *Harmonia axyridis*. **(A)** The soluble trehalase activity in *H. axyridis* after the injection of ds*TRE2-like*, ds*TRE2*, ds*TRE2s*, and ds*GFP*. **(B)** The membrane-binding trehalase activity in *H. axyridis* after the injection of ds*TRE2-like*, ds*TRE2*, ds*TRE2s*, and ds*GFP*. The third and fourth instar larvae were chosen as the targets for dsRNA injection. ^∗^*P* < 0.05 and ^∗∗^*P* < 0.01.

Compared with the control group injected with *dsGFP*, the soluble trehalase activity mostly exhibited significant (*P* < 0.05) or highly significant (*P* < 0.01) increase after interference with the *TRE2-like* gene. At the same time, *TRE2s* RNAi also showed a very significant increase in the molting stage (*P* < 0.01). After interference with the *TRE2* gene, there was a significant (*P* < 0.05) increase in the molting process of the third instar larvae. Others showed significant (*P* < 0.05) or highly significant (*P* < 0.01) reduction (Figure 3A). In the results of membrane-bound trehalase activity, we found that the activity at the molting stage was lower, and the d*sTRE2-like* developmental stage showed a decrease or a significant decrease. After 48 h of injection of *dsTRE2* or *dsTRE2s*, the fourth instar larvae were significantly (*P* < 0.05) or highly significant (*P* < 0.01) reduced (Figure 3B).

### Changes of Glycogen, Glucose, and Trehalose Content After RNAi

**FIGURE 4:**
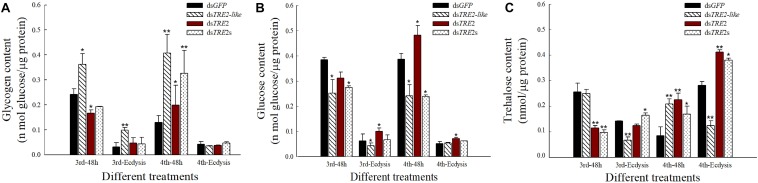
Content of glycogen, glucose, and trehalose in *Harmonia axyridis* after RNAi. **(A)** The glycogen content in *H. axyridis* after the injection of ds*TRE2-like*, ds*TRE2*, ds*TRE2s*, and ds*GF*P. **(B)** The glucose content in *H. axyridis* after the injection of ds*TRE2-like*, ds*TRE2*, ds*TRE2s*, and ds*GFP*. **(C)** The trehalose content in *H. axyridis* after the injection of ds*TRE2-like*, ds*TRE2*, ds*TRE2s*, and ds*GFP*. The third and fourth instar larvae were chosen as the targets for dsRNA injection. ^∗^*P* < 0.05 and ^∗∗^*P* < 0.01.

Compared with the control group injected with ds*GFP*, most of the glycogen content that interfered with *TRE2-like* showed a significant (*P* < 0.05) or highly significant (*P* < 0.01) increase (Figure 4A). After *TRE2-like* interference, most of the glucose content decreased or decreased significantly (*P* < 0.05). After interference with *TRE2s*, the third instar larvae and the fourth instar larvae also showed a significant decrease in interference for 48 h. After interference with *TRE2*, most of the glucose content increased significantly (*P* < 0.05) (Figure 4B). The interference of *TRE2-like* in the molting stage led to significant decrease in the trehalose content (*P* < 0.01). The trehalose content increased significantly (*P* < 0.01) during the 48th hour of *TRE2* and the molting of the fourth instar larvae. The change in trehalose content after injection of *dsTRE2s* was consistent with the interference with the *TRE2* gene, showing a significant increase (*P* < 0.05) (Figure 4C).

### Effects of RNAi on the Expression of *TPS*, *GS*, and *GP* Genes

**FIGURE 5:**
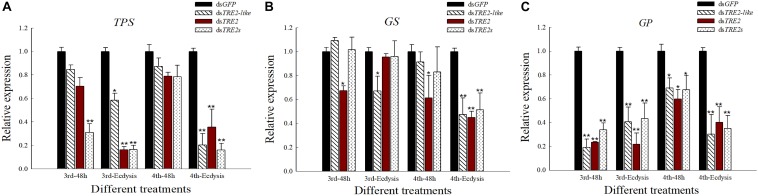
Effects of RNAi on the expression of *TPS*, *GS*, and *GP* genes in *Harmonia axyridis.* **(A)** The expression level of trehalose-6-phosphate synthase genes (*TPS*) gene in *H. axyridis* after RNAi. **(B)** The expression level of glycogen synthase (*GS*) gene in *H. axyridis* after RNAi. **(C)** The expression level of glycogen phosphorylase (*GP*) gene in *H. axyridis* after RNAi. The third and fourth instar larvae were chosen as the targets for dsRNA injection. ^∗^*P* < 0.05 and ^∗∗^*P* < 0.01.

Compared with the control group injected with ds*GFP*, the expression of trehalose-6-phosphate synthase (*TPS*) gene in *H. axyridis* showed a decrease after the injection of ds*TRE2-lik*e, ds*TR*E*2* or ds*TRE2s*. Particularly, the *TPS* gene expression in the third and fourth instar larvae showed a significant (*P* < 0.05) or highly significant (P < 0.01) decrease after RNAi (Figure 5A). With the injection of ds*TRE2-like*, ds*TRE2* or ds*TRE2s*, the expression of glycogen synthase (*GS*) gene in *H. axyridis* was decreased. Furthermore, after interference in the ecdysis of fourth instar larvae, all the injection treatment groups showed a significant (*P* < 0.05) or highly significant (*P* < 0.01) decrease (Figure 5B). After the injection of ds*TRE2-lik*e, ds*TRE2* or ds*TRE2s*, a significant (*P* < 0.05) or highly significant (*P* < 0.01) decrease of the expression of glycogen phosphorylase (*GP*) gene in all stages was recorded (Figure 5C).

### The Expression of *CHSA* and *CHSB* Genes When *TRE2-like* and *TRE2* Silenced

**FIGURE 6:**
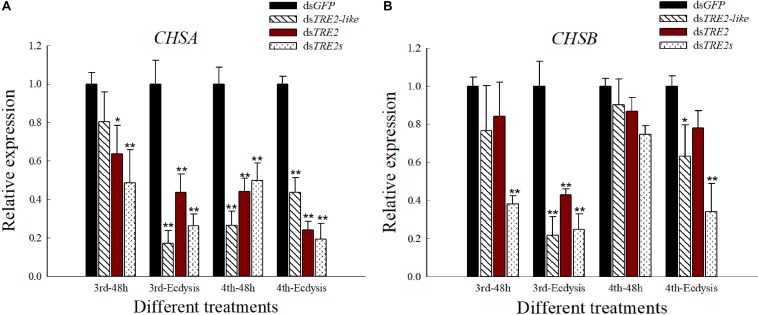
Effects of RNAi on the expression of *CHSA* and *CHSB* genes in *Harmonia axyridis*. **(A)** The expression level of chitin synthase gene A (*CHSA*) gene in *H. axyridis* after RNAi. **(B)** The expression level of chitin synthase gene B (*CHSB*) gene in *H. axyridis* after RNAi. The third and fourth instar larvae were chosen as the targets for dsRNA injection. ^∗^*P* < 0.05 and ^∗∗^*P* < 0.01.

Compared with the control group injected with ds*GFP*, the group injecting with ds*TRE2-like*, ds*TRE2* or ds*TRE2s* showed a significant (*P* < 0.05) or highly significant (*P* < 0.01) decrease in the expression of chitin synthase gene A (*CHSA*) gene in *H. axyridis* (Figure 6A). Meanwhile, the trend of the expression of chitin synthase gene B (*CHSB*) gene was similar to the *CHSA*, except that there was no significant changes appeared in the fourth instar larvae with the injection of ds*TRE2-like*, ds*TRE2* or ds*TRE2s* (Figure 6B).

### The Effect of *TRE2-like* and *TRE2* RNAi on *TRE1* Expressed Genes

**FIGURE 7:**
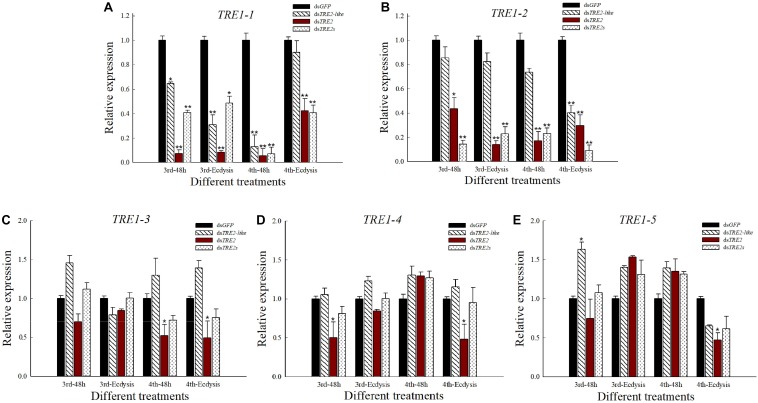
Expression level of *TRE1* in *Harmonia axyridis* after RNAi. **(A)** The expression level of *TRE1-1* gene in *H. axyridis* after RNAi. **(B)** The expression level of *TRE1-2* in *H. axyridis* after RNAi. **(C)** The expression level of *TRE1-3* in *H. axyridis* after RNAi. **(D)** The expression level of *TRE1-4* in *H. axyridis* after RNAi. **(E)** The expression level of *TRE1-5* in *H. axyridis* after RNAi. The third and fourth instar larvae were chosen as the targets for dsRNA injection. ^∗^*P* < 0.05 and ^∗∗^*P* < 0.01.

Compared with the control group injected with ds*GFP*, the expression of *TRE1-1* and *TRE1-2* genes in both the ds*TRE2-like*, ds*TRE2* and ds*TRE2s* injected groups showed significant (*P* < 0.05) or highly significant (*P* < 0.01) decrease (Figures 7A,B). After the injection of ds*TRE2*, the expression of *TRE1-3* gene in *H. axyridis* was decreased or significantly (*P* < 0.05) decreased (Figure 7C). The expression of the *TRE1-4* gene was significantly (*P* < 0.01) decreased at 48 h after the injection of ds*TRE2* in the third instar larvae and during ecdysis in the fourth instar larvae (Figure 7D). As for the expression of the *TRE5* gene, there was significant (*P* < 0.05) decrease with the injection of ds*TRE2* (Figure 7E).

## Discussion

At present, RNAi technology has been relatively mature used in a variety of insects, such as *Bombyx mori* (Ohnishi et al., 2006), and *T. castaneum* (Arakane et al., 2005). So, in this study, dsRNA of *TRE2-like* and *TRE2* was artificially synthesized *in vitro* and introduced into the *H. axyridis* by microinjection, to study the expression and function of *TRE2-lik*e and *TRE2* genes.

We have previously studied the *TRE2-like* and *TRE2* gene silencing in the pupal stage of the *H. axyridis* and found that the effects of interference were significant, and there were wing deformities after the RNAi (Zhang et al., 2019a). Similar findings were observed in the present study, wherein we found that silencing *TRE2-like* and *TRE2* genes seriously affected the whole nymph development process of *H. axyridis* leading to the inhibition of the growth of nymph or pupa, especially in the molting stage and further phenotypic abnormality and decreased survival rates (Figure 1). The results were similar to those obtained by inhibiting *TRE* gene expression in other insects, for example, phenotypic malformation occurs after interference with the *TRE2* gene in *S. exigua* (Chen et al., 2010) or *Brown planthopper* (Zhao et al., 2016). It has been confirmed that the deformed phenotype was caused by a decrease in the transcription level of trehalase (Tang et al., 2016; Zhang et al., 2017b). Therefore, we tested the expression levels of *TRE2-like* and *TRE2* genes after RNAi, the results show that the relative expression levels of *TRE2-like* and *TRE2* genes were significantly decreased (Figure 2). The result is consistent with the above conclusion. Moreover, in the present study, we found that the pupal weight reduced by RNAi and found that silencing of *TRE2-like* or *TRE2* genes have different effects on insect viability. In fact, the abnormal wings have been found owing to the significant decrease in the expression levels of chitin synthase and wing developmental network genes when *TRE* gene or TRE enzyme activity have been inhibited (Tang et al., 2017; Zhang et al., 2017a).

Trehalase is able to decompose the important energy storage material and the stress metabolite- trehalose in insects. The changes in gene expression and enzyme activity affect the life processes of insect molting, metamorphosis and reproduction (Tang et al., 2012). Studies have reported that sustained expression of *TRE2* gene and activity of TRE2 are necessary for the metabolism of hemolymph trehalose to meet the energy requirements of the midgut cells (Tatun et al., 2008). The results of this study showed that the interference of *TRE2-like* and *TRE2* genes decreased the activity of *TRE2* in *H. axyridis.* It is speculated that after inhibiting the expression of the *TRE2-like* gene, the activity of the trehalase activity will be reduced (Figure 3), which has already been confirmed in silkworm larvae (Mitsumasu et al., 2005). In addition, based on the results of this experiment, we speculate that *TRE2* gene may mainly regulate soluble trehalase activity, while *TRE2-like* gene tends to affect membrane-bound trehalase activity. But this view needs further confirmation and validation through other experiments. Trehalose and trehalase activities play a key role in regulating multiple physiological processes of insects (Ge et al., 2011). The decrease in trehalase gene expression and enzyme activity affects the rate of trehalose synthesis, resulting in insufficient energy, leading to wing deformity and stunting. Previous studies have shown that membrane-bound trehalase with higher activity is mainly present in the muscles of insects, hydrolyzing trehalose in food and providing energy for the movement and development of insects (Becker et al., 1996).

Trehalose has been well demonstrated in insect physiology as an energy source for insects, maintaining the glucose level (Santos et al., 2012). Studies have shown that the *TRE* gene affects the content of three sugars by regulating gene expression and enzyme activity (Santos et al., 2012; Shi et al., 2016). In the present study, after the successful interference of *TRE2-like* gene the activity of membrane-bound trehalose and the trehalose content were reduced and the glucose levels also declined (Figures 4B,C). Related reports have shown that when trehalose hydrolysis into glucose is inhibited, it affects the energy level and blood glucose content in various cells of insects, and thus affecting other physiological pathways (Chen et al., 2010). Meanwhile, we found that after the interference of *TRE2-like* gene, the glycogen content was increased (Figure 4A), it has been indicated that trehalose and glycogen are important energy storage substances in insects as well as play an important role in energy metabolism of insects (Tan et al., 2014). The increase in the glycogen content indicates that trehalose or glucose is decomposed into glycogen to ensure normal physiological activities.

Glycogen metabolism requires two enzymes, named *GS* gene for the synthesis and *GP* gene for the decomposition, and both these enzymes are controlled by hormones and play a key role in glycogen metabolic balance (Łopieńska-Biernat et al., 2019). In this study, we investigated the glycogen metabolism, after the injection of ds*TRE2-lik*e, the expression of *GP* gene showed decrease at the mRNA level and the *GS* gene expression decreased in the ecdysis stage (Figures 5B,C). Glycogen is converted into trehalose to increase the blood sugar concentration of insects and adapt to the adverse environment. *GP* gene can decompose glycogen into glucose-1-phosphate, which can then interact with uridine diphosphate (UDPG) and be converted into trehalose (Tang et al., 2018). The null mutants of *GS* and *GP* genes displayed growth defects and larval lethality indicating that glycogen plays a crucial role in larval development (Yamada et al., 2019).

In insects, trehalose is first synthesized by *TPS*, which is degraded to glucose by trehalase when demanding for energy supply (Tang et al., 2018). In the present study, we found that silencing *TRE2-like* and *TRE2* genes reduced the relative expression of *TPS* genes, and caused the phenomenon of wing deformity (Figure 5A). Studies have shown that silencing *TPS* inhibits the expression of chitin biosynthetic pathways and lipid catabolism-related genes, thereby affecting the development of insect (Chen et al., 2018). Previous studies have shown that increased expression of *TPS* gene can increase anti-stress ability and protect cell structure. In addition, when the expression of *TPS* gene was severely inhibited, abnormal phenotypes exhibited, especially molting and wing deformities, as well as the increased mortality (Tang et al., 2016).

In addition to providing energy for the growth and development of insect, as the first key enzyme in the chitin synthesis pathway, trehalase is also closely related to the formation of insect chitin (Chen et al., 2010). In insects, trehalose synthesized by *TPS* is the main substrate for chitin biosynthesis (Shukla et al., 2015), and *TPS* gene can effectively regulate the biosynthesis or degradation of chitin by *TRE* gene. This hypothesis has been confirmed by knocking down the *TPS* genes in *N. lugens*, in which some genes involved in the chitin biosynthetic pathway and almost all *CHS* genes have decreased expression (Yang et al., 2017). In this study, silencing *TRE2-like* and *TRE2* not only reduced the expression level of the target gene, but also reduced the relative expression levels of *TPS*, *CHSA*, and *CHSB* genes (Figure 6). Studies have shown that the synthesis of new epidermal chitin in migratory locust requires a large amount of trehalase, and the silencing of the gene mainly affects the synthesis of chitin in insect epidermis (Liu et al., 2016). Furthermore, research shows that insects renew the stratum corneum of the exoskeleton by degrading the chitin and cuticle proteins and synthesizing a new cuticle during ecdysis, which is a necessary process for the normal growth and development of insects (Merzendorfer and Zimoch, 2003; Deng et al., 2016; Ye et al., 2019). The function of the trehalase-encoding gene in the synthesis of chitin in *Brown planthopper* using RNAi technology showed that after inhibiting the expression of the trehalase-encoding gene, chitin synthase-encoding gene and other genes were also down-regulated correspondingly. The inhibition of chitin deacetylase expression can affect the ecdysis of larval-to-pupa and pupa-to-adult in *Leptinotarsa decemlineata* (Wu et al., 2019). These results indicate that once the supply balance of trehalose metabolism in insects is broken, it will directly affect the chitin synthesis of insects.

It is well known that the *H. axyridis* has five soluble trehalases and two membrane-bound trehalase genes. So, to further understand the function of the membrane-bound gene, we examined the relative expression levels of the five *TRE1* genes after RNAi *TRE2-like* or *TRE2*. From the results, the effect of interference *TRE2-like* or *TRE2* on *TRE1-1* gene expression level was significant, indicating that *TRE2-like*, *TRE2*, and *TRE1-1* may have similar functions. In addition, the interference of *TRE2* gene significantly affected the expression of *TRE1-1* and *TRE1-2* genes levels, but had no significant effect on *TRE1-3*, *TRE1-4*, and *TRE1-5*, indicating *TRE2* and *TRE1-1* and *TRE1-2* has a higher functional similarity and a greater influence on each other (Figure 7). The results are consistent with the relative expression changes of three trehalase genes in *brown planthopper* (Zhao et al., 2016). How to play the biological control role of *H. axyridis* depends on how to produce and put into use on a large scale. However, there is still no way to coordinate the production input of the *H. axyridis* with the pest outbreak time. Therefore, through the study of *TRE* molecular mechanism of *H. axyridis*, this experiment laid a foundation for the production of natural predators of *H. axyridis* and realized its biological control effect better.

## Conclusion

In conclusion, the results revealed that RNAi can effectively reduce the expression of the target genes. The injection of ds*TRE2-like* and ds*TRE2* into *H. axyridis* can disrupt the metabolism of trehalose in the body and affect the supply of glycogen and glucose, causing difficulty in the synthesis of chitin, consequently leading to wing deformity. The results lay a foundation for exploring the potential functions and regulatory mechanisms of insect *TRE2*. Therefore, it is particularly important to further study the molecular mechanism of trehalose in other insects.

## Data Availability Statement

Publicly available datasets were analyzed in this study. These datasets can be found here: TRE2-like: KX349224.1 and TRE2: KX349225.1.

## Author Contributions

BT and SW conceived and designed the manuscript structure. YL, XC, S-SW, B-YP, and S-GW performed the current articles collection and trehalose metabolism genes’ analysis. YL and S-SW wrote the manuscript.

## Conflict of Interest

The authors declare that the research was conducted in the absence of any commercial or financial relationships that could be construed as a potential conflict of interest. The reviewer SN declared a past collaboration with one of the authors BT.

## Supplementary Material

IMAGE S1 Nucleotide and deduced amino acid sequences of *Harmonia axyridis* membrane-bound-like trehalase (TRE2-like). Initiation and termination codons are indicated by bold typeface and italic. The conserved domains used for dsRNA synthesis of *TRE2-like* (751–1362 bp) is shaded in gray. qRT–PCR primers sequences used to detect TRE2-lik is indicated by bold typeface and red-colored shading.

IMAGE S2 Nucleotide and deduced amino acid sequences of *Harmonia axyridis* membrane-bound trehalase (TRE2). Initiation and termination codons are indicated by bold typeface and italic. The conserved domains used for dsRNA synthesis of *TRE2* (1897–2308 bp) is shaded in gray. qRT–PCR primers sequences used to detect TRE2-lik is indicated by bold typeface and red-colored shading.
