# Supplementary figures and images for: Evaluation of the Expression and Function of the TRE2-like and TRE2 Genes in Ecdysis of Harmonia axyridis
Source: Front Physiol. 2019 Nov 1;10:1371. doi: 10.3389/fphys.2019.01371 (PMC6839538; doi:10.3389/fphys.2019.01371)

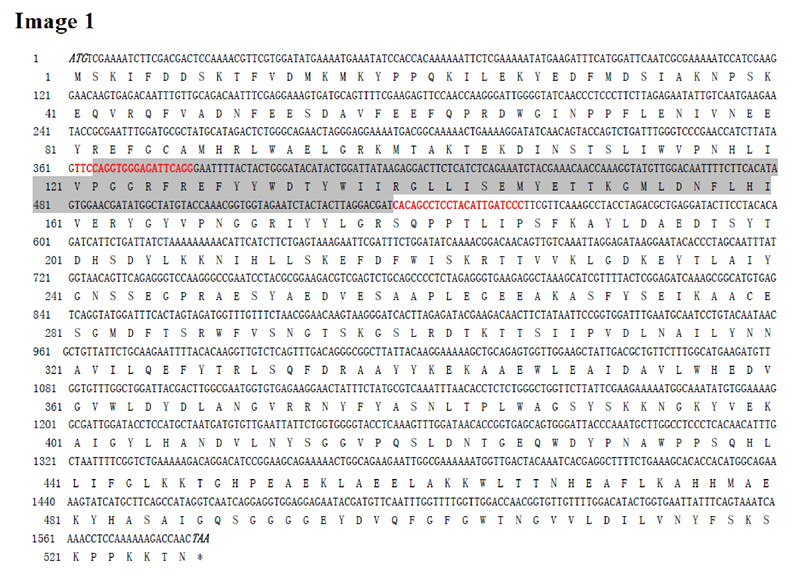

Supplement: IMAGE S1 — Nucleotide and deduced amino acid sequences of Harmonia axyridis membrane-bound-like trehalase (TRE2-like). Initiation and termination codons are indicated by bold typeface and italic. The conserved domains used for dsRNA synthesis of TRE2-like (751–1362 bp) is shaded in gray. qRT–PCR primers sequences used to detect TRE2-lik is indicated by bold typeface and red-colored shading. [file Image_1.tif]

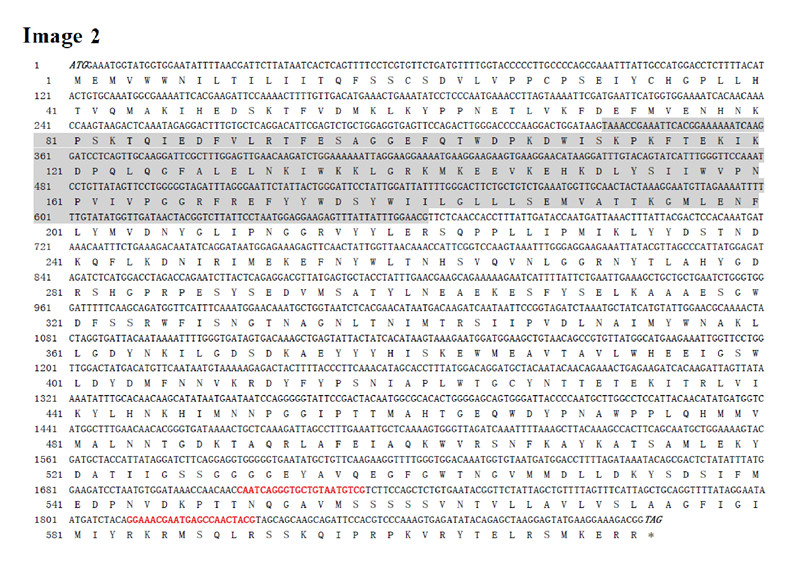

Supplement: IMAGE S2 — Nucleotide and deduced amino acid sequences of Harmonia axyridis membrane-bound trehalase (TRE2). Initiation and termination codons are indicated by bold typeface and italic. The conserved domains used for dsRNA synthesis of TRE2 (1897–2308 bp) is shaded in gray. qRT–PCR primers sequences used to detect TRE2-lik is indicated by bold typeface and red-colored shading. [file Image_2.tif]
